# An analysis of the ethics of lockdown in India

**DOI:** 10.1007/s41649-020-00133-3

**Published:** 2020-07-09

**Authors:** Meghna Ann Arunachalam, Aarti Halwai

**Affiliations:** YU-FIC Research Ethics Master’s Program for India, Yenepoya Deemed to be University, Mangalore, India

**Keywords:** Lockdown, India, Vulnerable, Justice, Community engagement, Restrictive measures

## Abstract

Over the past 6 months, coronavirus-induced disease (COVID-19) has spread across 212 countries, affecting millions of people. As it has no known cure, social distancing is highly recommended for prevention of spread of the disease. Here, we have described the impact of the social distancing measures implemented by the Government of India on various sections of the society, especially the vulnerable sections. Furthermore, we have presented an analysis of these measures, according to the World Health Organization´s Guidance for Managing Ethical Issues in Infectious Disease Outbreaks (2016); we have also applied principles, as applicable, from the Indian Council of Medical Research’s National Ethical Guidelines for Biomedical and Health Research Involving Human Participants (2017). Finally, we have presented several measures that should have been adopted before and in addition to implementing the lockdown to improve its effectiveness.

## Introduction

Several cases of an atypical pneumonia were first reported in December 2019 in Wuhan, China (Spiteri et al. 2020). The disease, caused by a novel coronavirus believed to originate from a seafood market in Wuhan, spread through close contact and eventually resulted in a pandemic (involving more than 212 countries, with an estimated mortality rate of 3.4%). The pathogen and disease were officially named severe acute respiratory syndrome coronavirus 2 (SARS-CoV-2) and coronavirus-induced disease 2019 (COVID-19) (World Health Organization 2020). As of 7 June 2020, almost 7 million cases and 402,561 deaths have been reported worldwide, and the numbers continue to rise (Worldometer 2020a). Being a novel virus, no therapy or vaccine exists; therefore, the focus is mainly on containment of the virus and prevention of the disease.

## Situation in India

The first case of coronavirus in India was identified on 30 January 2020. By 3 February, the number of cases increased to 3. On 4 March, 22 new cases were identified, of which 14 were from a group of tourists who had arrived from Italy (The Week 2020). In March, India also reported its first coronavirus-related death. The number of confirmed cases in India crossed 1000 on 29 March, 30,000 on 28 April, and 180,000 on 30 May. The death toll crossed 50 on 1 April, 1000 on 28 April, and 5000 on 30 May. As of 7 June 2020, the numbers of infected cases and deaths are 247,195 and 6950, respectively (Worldometer 2020b).

Various measures have been implemented globally to restrict community spread of the virus; these include isolation, encouraging social distancing, quarantine, testing, tracing contacts of positive cases, and sealing of borders; frequent hand sanitization and use of masks are also highly recommended. India began thermal screening of passengers arriving from China on 21 January (Economic Times 2020a). In February, the screening was extended to passengers from Thailand, Singapore, Hong Kong, Japan, and South Korea (India Today 2020a), and the list swelled to include Nepal, Vietnam, Indonesia, and Malaysia by the end of February (Outlook 2020). By 3 March, the Indian Government had stopped issuing new visas (Bureau of Immigration 2020), and all Indian nationals returning from COVID-affected countries were quarantined for 14 days (Al Jazeera 2020). Several ministries were tasked with providing essential goods and services during the outbreak (Economic Times 2020b). Seven ministries were involved in setting up treatment and quarantine facilities across the country (Mishra 2020). On 17 March, the Government of India advised the states to adopt social distancing measures. Furthermore, a COVID-19 Economic Response Task Force was formed (DD News 2020). Travel restrictions were also imposed, and borders were sealed. By mid-March, all schools and educational institutions in the country were closed (Sharma 2020). On 22 March, a curfew was announced for the benefit of the people (*Janata* curfew), wherein they were advised to stay at home. This was followed by a lockdown in all districts where positive cases had been reported. A 21-day national lockdown was announced on 24 March until 14 April (First Post 2020); this was further extended until May 03 (Bhaskar 2020). A third lockdown extending until 17 May was announced on 1 May (India Today 2020b). This was followed by a fourth lockdown till 31 May 2020, and a fifth lockdown (for containment zones only), which is ongoing (The Hindu 2020a).

Although employed to save lives, these methods have increased the struggle for many of India’s citizens, especially the poor and marginalized communities. The lockdown measures have caused extensive damage and destroyed many lives and livelihoods, leading to an ethical and economic crisis.

India has some of the most densely populated cities in the world. Mumbai, one of the largest cities in India, is also home to the biggest slum in Asia. Here, social distancing is practically impossible. Furthermore, these cities attract large numbers of migrants, who move to cities for a better livelihood, often living in tenements or *chawls* under unsanitary conditions; the rooms in these *chawls* are 8 × 10′ in size and house up to 6 members (Chaitanya and Naik 2020). For most of these migrants, working as daily wage laborers, life came to a standstill once the lockdown was announced. Millions were stranded in cities with no source of livelihood. Many were evicted from their rented accommodations. Furthermore, once the train and bus services were suspended and borders were sealed, they had no means of transport. This forced them to walk several hundred kilometers to their hometowns and villages. Although the authorities tried to arrange transport and accommodation, it was too little, too late. Many could not stand the strain of this arduous journey and died en route (Huffpost 2020). Many were beaten by the police at the borders; they were further indignified by being sprayed with disinfectant (Datta 2020). Millions of people in cities, even in the lower and upper middle class, lost their jobs; several micro, small, and medium enterprises were forced to shut down (Yadav 2020). The lockdown has restricted consumption, thus affecting the supply and demand chain. This has in turn affected the employment prospects for the people. Consequently, there are no jobs and the economy has been adversely affected.

While the plight of unorganized sector in slums and of labor population is bad, the situation in rural India is no better. Farmers face drastic economic losses as there are no buyers to purchase their produce owing to the lockdown. Many of them rely on selling their crops to repay their debts. However, in the current situation, there are no laborers to help harvest the crops and no demands for their produce (Gulf News 2020). Many have abandoned their crops by the roadside, as they have no means to transport them to the cities (The Hindu 2020b). Farmers have been known to burn their crops out of sheer desperation (The Hindu 2020c).

At a personal level, the lockdown has forced people to remain in their houses to restrict the spread of the virus, without taking into consideration their daily needs. Many are facing issues, such as inadequate supply of food, groceries, and medicines. Originally, the borders were sealed even for ambulances and patients. This has now been relaxed (Economic Times 2020c), but the relaxation came after the loss of human life (New Indian Express 2020). Moreover, to prevent crowding at hospitals, the Government has attempted to implement telemedicine on a mass scale (Times of India 2020). However, most of India’s population does not have access to smartphones or the Internet, and many are unable to navigate the complicated web portals to access healthcare. Although 74% of the Indian population is considered literate, almost half this number have had less than 7 years of formal schooling (National Sample Survey Office 2016). This should have been considered before taking such steps.

Quarantine and isolation measures were implemented without taking into consideration the needs of the individuals. Several quarantine facilities lacked basic amenities, thus encouraging many to flee quarantine centers, defeating the entire purpose of the social distancing and quarantine.

## An analysis of the ethics of lockdown

As COVID-19 is highly contagious, with no established cure, social distancing measures are necessary. However, the high population density and poor socioeconomic conditions in India do not allow easy implementation of social distancing and good hygiene practices. Employing restrictive measures under these conditions is fraught with ethical issues. In this section, we aim to analyze the lockdown, as it was implemented in India, according to the World Health Organization’s Guidance for Managing Ethical Issues in Infectious Disease Outbreaks (2016); we have chosen to analyze the ethical issues in the COVID-19 scenario using these guidelines, because they are more comprehensive than most other guidelines and are also globally accepted. We have also applied principles, as applicable, from the Indian Council of Medical Research’s National Ethical Guidelines for Biomedical and Health Research Involving Human Participants (2017).

### Planning

Containment measures for epidemics in India are based on the Epidemic Diseases Act (1897). However, this Act is more than a century old. It consists of the following four sections: title and the extent, the powers of the state and central Government in implementing measures to contain the disease spread, penalties for violation of the measures, and legal protection to the implementing officers. This Act lacks description of the duties of the government and rights of the citizens, and a human rights perspective. Moreover, as public health is the responsibility of the state, different states follow different public health Acts; this leads to a lack of coordination in scientific responses in tackling the outbreak. They are also mostly “policing” in nature and deal with controlling and not preventing the outbreak. Other factors that need to be taken into account in the current scenario (which are lacking in the Epidemic Diseases Act of 1897) are increased urbanization, migration for livelihood, increased population density, extensive air travel as opposed to sea travel, ecological changes, and biosafety lapses (Rakesh 2016). By biosafety lapses, we refer to lapses that may occur during the collection, handling, and storage of infectious samples. Considering all of the above, the Epidemic Diseases Act of 1897 is not an appropriate yardstick for ethical analysis of the measures implemented by the Indian Government in the COVID-19 outbreak.

For an appropriate ethical analysis of these measures, we need to view it through the lens of public health ethics. In addition to the four basic principles of ethics (autonomy, beneficence, non-maleficence, and justice), public health ethics also includes the principles of social justice, solidarity, reciprocity, and accountability and transparency (Indian Council of Medical Research 2017). Before employing any restrictive measures, the Government should have performed a risk-benefit analysis, including an assessment of the long-term effects on society and the economy, potential harms, and effects on the vulnerable sections of the society. Although the pandemic spread very rapidly, India was affected quite late, and proactive steps could have and should have been taken by the Government. The authorities could have made better arrangements for the vulnerable sections of the society. WHO defines vulnerability as the degree to which a population, individual, or organization is unable to anticipate, cope with, resist, and recover from the impacts of disasters (Wisner and Adams 2002). Social distancing is possible for those with a place to stay, and restrictive measures can be applied to those with adequate food and water supply. The loss of daily wages and accommodation and lack of modes of transport increased vulnerability in these already vulnerable sections of the society, violating the principle of justice. People who were economically backward have been left economically and emotionally destitute (Pandey 2020). According to Rawls’ (1971) difference principle, economic inequalities can be tolerated only if they make the least advantaged sections of society as well off as they could possibly be. The lockdown has resulted in quite the reverse. Although it may have protected people from COVID-19, it nevertheless caused more than 300 deaths (reported) from starvation, exhaustion, suicide, and other lockdown-related causes (The Hindu 2020d).

Several renowned bioethicists and philosophers consider autonomy to be primus inter pares among the four ethical principles. First, no form of ethics or morality is possible without respect for autonomy or right to self-determination; second, a fundamental requirement for the fulfilment of other principles, like beneficence and non-maleficence, is respect for autonomy (Gillon 2003). Although the WHO Guidance for Managing Ethical Issues in Infectious Disease Outbreaks (2016) allows restrictive measures to be applied in case of pandemics for the benefit of the community, the restrictions should be least infringing; this is also advocated in the Siracusa Principles on the Limitation and Derogation Provisions in the International Covenant on Civil and Political Rights (American Association for the International Commission of Jurists 1985). Furthermore, certain guidelines should be followed in employing these restrictive measures. The abovementioned WHO guidelines stress on the obligations of the Government toward improving the social and environmental conditions, and the health system and accessibility. They also discuss the importance of engaging in public health surveillance and preventive activities. It is pointless advocating frequent hand sanitization to people who may not have access to clean water or to villagers who have to walk miles to fetch water. Moreover, people who live on the footpaths or in slums cannot practice social distancing. The daily wage earners were also adversely affected by the lockdown. If people’s autonomy was to be restricted, the Government should have exercised reciprocity. Arrangements should have been made to provide people with a place to stay, clean water, and adequate rations. Lockdown implementation dates should have been announced well in advance allowing people to prepare for the lockdown. Although travel might have increased the spread of the disease, migrants should have been allowed to return to their villages instead of starving on the streets in cities or walking several hundred miles to their villages. The authorities could also have assisted in making necessary arrangements for farmers to harvest and transport their produce.

### Community engagement

The guidelines also highlight the importance of community engagement, from the initial stage through the decision-making and implementation, in understanding the perspectives, needs, and requirements of various sections of the society. Community engagement encourages compliance with the lockdown, and this compliance is reflective of the trustworthiness of the system. However, in India, no two-way dialogue was facilitated. Most of the population was unaware of the gravity of the situation. Furthermore, there was no involvement of the vulnerable sections of the society, who were adversely affected by the lockdown, in the decision-making process. Had arrangements been made to involve the community, explain the magnitude of the problem, address their requirements, and respect their autonomy, compliance with the lockdown would likely have been better. The implementation of the lockdown clearly lacked transparency and accountability.

### Implementation

The guidelines put forth the need for additional safeguards for particularly vulnerable groups. In the current scenario, this also includes the senior citizens. Not only are they particularly vulnerable to the virus but also to other factors. They may be old and living alone; many also suffer from various ailments requiring regular visits to the hospital and nursing care. For them, an adequate supply of food, medicines, and regular access to the hospital become a necessity.

According to the guidelines, the effectiveness of restrictive measures, including isolation, quarantine, travel advisory or restrictions, and community-based measures to reduce contact between people, lies in their acceptance by society, which would again require societal involvement in the decision-making and implementation. Furthermore, implementation of lockdown should be equal for all; it should not differ for some based on their social, political, or economic status, because disobedience by even a few would pose a risk to the rest of the community. In India, while the authorities and police employ stringent measures with the vulnerable sections (like the migrants who were sprayed with disinfectants), people of certain affiliations have been able to conduct marriages and other ceremonies (Economic Times 2020d). This violates the principle of social justice.

The guidelines also suggest collaborative networking with other nations affected by the pandemic to understand and learn from their experiences, needs, and the measures employed. A very important lesson can be learnt from how Kerala, a very small state in India, by using a framework of equality, social rights, and public trust, has managed to successfully combat this outbreak. The first few recorded cases of COVID-19 in India occurred in Kerala. However, as of 7 June 2020, the overall mortality rate in India is 2.81% (number of confirmed cases, 246,628; number of deaths, 6929); however, in Kerala, the mortality has been as low as 0.83% (number of confirmed cases, 1807; number of deaths, 15) (Government of India 2020). Kerala’s proactiveness, good healthcare system and contact tracing initiative, appropriate resource allocation, and good community involvement fostered public trust and compliance, allowing them to overcome the situation better and faster than the rest of the nation (Biswas 2020). In addition to a good healthcare system, Kerala also has good ground-level democracy (*Panchayat* system). The panchayat system in India refers to self-governance; it is a system followed at grass-root levels in rural areas. The village panchayats are basic units of the local administration. All posts at the panchayat level in Kerala are filled and have been actively working in a collaborative manner in the panchayat-initiated programs, such as “Ashraya” (which deals with feeding the destitute). These programs were initiated and are being managed by the panchayats, and this has also facilitated them in dealing with the COVID-19 pandemic (Raghunandan 2020).

## Conclusion

Although the social distancing measures implemented by the Government of India were partly effective in preventing the spread of the disease, large sections of the population have been adversely affected in the process. Many have been rendered homeless and left stranded in the cities, and the economy has also taken a setback. People who avoided COVID-19, in many instances, succumbed to starvation and the indignity of begging. Ethical implementation of these measures could have made them more effective. Furthermore, social distancing measures have only bought time. COVID-19 is here to stay. An important issue to consider is whether at the end of the lockdown period, our healthcare system will be able to bear the onslaught of COVID-19 victims. Will the gain be proportional to the harm caused by the lockdown? Only time will tell.
